# Effectiveness of Metacognitive Regulation Intervention on Attention-Deficit–Hyperactivity Disorder Students’ Scientific Ability and Motivation

**DOI:** 10.3389/fpsyg.2021.747961

**Published:** 2021-12-23

**Authors:** Haoyuan Zheng, Yang Dong, YuanKe Sun, Jie Yang, Chongbo Yuan, JinDao Wang, Weiyang Dong

**Affiliations:** ^1^Department of Teacher Education, Guangzhou Huashang College, Guangzhou, China; ^2^Research Center for Overseas Studies and Media Reports on Hainan, Hainan University, Haikou, China; ^3^Department of English, Hainan University, Haikou, China; ^4^Department of Science and Environmental Studies, Education University of Hong Kong, Hong Kong, Hong Kong SAR, China; ^5^Department of Chinese Studies, Hong Kong Open University, Hong Kong, Hong Kong SAR, China

**Keywords:** metacognition, ADHD learners, regulation effect, dynamic interaction model, astronomy, primary school

## Abstract

This study investigated the effect of metacognitive regulation (McR) intervention on attention-deficit–hyperactivity disorder (ADHD) students’ astronomy knowledge acquisition and learning motivation. Through a cognitive-behavioural treatment design, this study selected 97 ADHD learners who had poor academic performance. This study divided ADHD students randomly into one experimental group and one control group. After 15 weeks of intervention, results showed that the experimental group students performed significantly better than the control group in scientific abilities, learning motivation, and metacognition. Results suggested that the McR intervention is an effective approach for improving the ADHD students’ science knowledge learning abilities.

## Introduction

Metacognitive regulation (McR) refers to an ability to modify cognitive processes and strategies to remain in control of their learning conflicts; it describes how students monitor and control their knowledge acquisition process (Zimmerman, 1995; Fernandez-Duque et al., 2000; Efklides, 2008). These McR abilities are implemented in McR activities and further comprised more concrete regulation strategies, such as presented task analysis, learning content orientation, task perception awareness, task problem-solving decomposition, progress monitoring, collaboration monitoring, evaluating learning outcomes, and evaluating the learning process. McR activities depend on the regulative agents involved and their underlying intentions, which include students’ individual learning, collaborative learning with classmates or peers, or based on a group collaborative learning process (Lajoie and Lu, 2012; Rogat and Adams-Wiggins, 2014; De Backer et al., 2015). Insufficient development of McR may generate the students’ knowledge misconception in a science discipline (Greene and Azevedo, 2010; Khosa and Volet, 2014; Ucan and Webb, 2015) and learning difficulties (Hurme et al., 2009; Jokić and Whitebread, 2011; Dragan, 2015; Goudas et al., 2017) and decrease the students’ learning motivation (Paris and Winograd, 1990; Sungur, 2007; Zimmerman and Moylan, 2009).

Previous studies have shown that the students’ awareness of McR can also be raised through observation of the modelled McR during peer interactions within students (e.g., collaborated learning) (De Backer et al., 2015; Raes et al., 2016), individual personal self-checking by providing McR rubrics (Papaioannou et al., 2012), and team-based learning supervised by an instructor (De Backer et al., 2016). The current literature has demonstrated the value of team-based learning when supervised by an instructor at the McR intervention (Schraw et al., 2006; De Backer et al., 2016; Hadwin et al., 2017). During this format intervention, previous studies report that students explicitly felt the need to regulate the learning interactions amongst instructors, peers, presented knowledge, and the learning processes taking place because they are reminded or required to engage in the collaborative goal set by the instructor and attend conceptual discussions with peers or groupmates, to control their own comprehension and check on learning outcomes collaboratively (De Backer et al., 2016; Hadwin et al., 2017).

Metacognitive regulation is easier to be trained and developed in an early school age than later in the school career (Kuhn, 2000). The previous cognitive apprenticeship paradigm suggests that the students’ McR awareness can emphasise learning through guided practice (Collins et al., 1988). Previous studies demonstrate that the key approach to develop the students’ McR awareness is to promote prompting the evaluation of their performance, compared with expected learning outcomes (Hadwin et al., 2005). Students can be challenged to internalise the expected behaviour at the individual level, which requires regulative practice in settings (De Backer et al., 2016). Moreover, an interactive learner–teacher learning format can be established, which prompts students to clarify, control, judge, and regulate their learning, which is aimed at consolidating metacognitive knowledge and skills (Schunk and Zimmerman, 2007).

Previous studies have shown that McR treatments improved the students’ performance in mathematics (Desoete et al., 2003; Bol et al., 2016; Vula et al., 2017), science (Abd-El-Khalick and Akerson, 2009; Peters and Kitsantas, 2010; Zepeda et al., 2015), and engineering (Clancey, 1988; Vrugt and Oort, 2008). Astronomy knowledge, which provides great learning resources on the students’ scientific ability development (e.g., spatial thinking and mathematical thinking skills’ application), has been less concerned. Astronomy knowledge in primary school introduces relevant concepts and knowledge in terms of celestial body units (e.g., Earth, Sun, and Moon). Astronomy knowledge has unique characteristics in typical knowledge integration (e.g., integrating mathematics and geography) and contributes to the learners’ problem-solving abilities. Moreover, students can learn the principles of natural laws easily in learning astronomy. Astronomy knowledge not only provides typical natural knowledge to students, but also offers great potential scientific thinking opportunities. However, only a few studies have explored the McR intervention effect in astronomy knowledge learning.

Students who have ADHD symptoms usually experience more disruptive and off-task behaviours than typical developing peers (Tamm et al., 2014; Pezzica et al., 2018). Studies have demonstrated that ADHD students have experienced high levels of learning stress and may have an increased risk for mental health disorders (Evans et al., 2020). Behavioural interventions are promising as ways to improving attention span and impulsivity in the development of academic performance (e.g., ability, learning motivation). Extensive studies have applied intervention designs to address learning problems (e.g., attention difficulties) for ADHD students. For example, the cognitive-behavioural treatment design, including knowledge translation or clarification and phenomena reason elaboration, has been confirmed as an effective approach to promote the ADHD learners’ academic performance (Hasson-Ohayon, 2012; Pöttgen et al., 2015; Moritz et al., 2019). Moreover, previous studies have shown that group learning will benefit more ADHD learners than individual independent learning (Tamm et al., 2014; Pezzica et al., 2018). Previous studies have also confirmed that the teacher-mediated instructional approach is more effective than the printed words instructional approach on the ADHD students’ cognition treatment (Hacker et al., 2019). McR emphasise cognitive control during the learning progress. However, the McR approach benefits on the ADHD students’ attention defect and academic performance remains unclear.

The dynamic interactional model (DIM; Toglia, 2018) in learning emphasises that learning is a continuous product of the dynamic interaction between the individual, task, and environment. Under the science education context, the use of McR can be promoted through a wide range of meaningful activities by improving activity management with strategy intervention (Josman and Regev, 2018; Toglia, 2018). However, whether the statement on DIM can be extended for ADHD students remains unknown, especially in the astronomy knowledge learning context.

### The Current Study

To address the aforementioned problems, this study aims to investigate the effect of McR intervention on the primary school ADHD students’ academic performance in the astronomy knowledge domain. Within the astronomy learning context, this study implements the McR intervention to primary school ADHD students, exploring the possible improvements in the ADHD students’ metacognition, learning motivation, and scientific ability, thus applying the DIM statement in ADHD students’ behaviour treatment in an astronomy learning context. The following research questions and correspondence hypotheses are listed as follows:

*Research question 1*: Does McR intervention significantly improve the ADHD students’ scientific ability?
*Research question 2*: Does McR intervention enhance the ADHD students’ science learning motivation?
*Research question 3*: What is the effect of McR intervention on the ADHD students’ metacognition development?

## Materials and Methods

### Participants

This study recruited 97 ADHD grade 5 primary school students from one typical primary school in Chengdu, China. All 97 students fulfilled the full DSM-IV criteria for ADHD at the age of 10, which were included into this study. The DSM-IV home version is a caregiver-reported rating scale that has been widely used in ADHD studies of primary school children (Roberts et al., 2019; Pang et al., 2021). The scale has strong discriminant validity both within the ADHD subtypes and between children with ADHD and without ADHD. The psychometric properties of the Chinese version of this scale also have been validated for the use amongst children aged 6–17 years in China (Su et al., 2015). The ADHD DSM-IV asks the child’s primary caregiver (kids’ teacher or guardian) to rate the frequency of 18 ADHD symptoms that occurred over the past 6 months. The primary caregiver is defined as the students at home or school most often responsible for the student’s care, typically the mother or paternal. Symptoms were rated on a four-point Likert scale, for which 0 = rarely or never, 1 = sometimes, 2 = often, and 3 = very often. To easily interpret the results, we use average scores to report the result, and scores for each item were then summed to reach a total score that ranged from zero to 3 points. Previous studies have found that a cut-off point of 2 yields optimal sensitivity and specificity in distinguishing children at risk of ADHD amongst students in urban China (Su et al., 2015; Tong et al., 2018). Following these methods, we similarly consider children with average scores above 2 to be at risk of ADHD. All participants should be diagnosed with at least four symptoms of inattention or hyperactivity (with a rating of “2” or “3,” mean = 2.62, SD = 0.17) on the DSM-IV rating scale (Nigg, 1999). To lower down the risk of ADHD diagnosis, clinical interviews were applied by trained medical centre health care professional officers at students’ school. Participants received neuropediatric or psychiatry services at school medical centre every year. In addition, participants received new round neuropediatry or psychiatry services at school medical centre 1 month ago before the intervention. All participants were ADHD students without any other comorbid problems (e.g., listening difficulties). The reason why we selected Grade 5 students as participants is that all Grade 5 students have the basic required learning experience in science knowledge, ensuring that the learning content will not be overloaded. All students had poor academic performance in Chengdu’s standard multidiscipline examinations. Moreover, all students had poor performance in science subjects and all these students came from low-income families. The 97 ADHD students were randomly assigned into one experimental group (EG) and one control group (CG). As a result, the EG had 49 ADHD students (25 boys, 24 girls, mean age = 9.74, *SD* = 0.45) and CG had 48 ADHD students (24 boys, 24 girls, mean age = 9.84, *SD* = 0.42). The difference analyses (one-way ANOVA and chi-square) showed that no significant differences exist amongst the ADHD students (EG and CG) in the standardised test of scientific abilities in astronomy [*F*(1,96) = 1.17, *p* > 0.10], non-verbal intelligence [*F*(2,151) = 0.46, *p* > 0.10], age [*F*(2,151) = 1.37, *p* > 0.10], and gender distribution [χ^2^ (2,151) = 1.09, *p* > 0.10], ensuring that the minimum benchmark of EG and CG was satisfied. No students have quit from the study during the intervention period. All students attended more than 80% of the intervention sessions.

### Research Procedure

The intervention design and measurements were reviewed by two Chinese science scholars and two primary school science teachers to ensure the teaching content validity before the implementation of the intervention programme. The curriculum teaching design was refined based on the feedbacks from both scholars and science subject teachers.

The selected measurement parameters (consistent reliability index and confirmatory factor analysis) of the final version, which was applied to the main study, are reported in Table 1. This study followed the major analysis and statistical tools from the confirmatory factor analysis: chi-square statistics, comparative fit index (*CFI*), the Tucker–Lewis index (*TLI*), root mean square error of approximation (*RMSEA*), and standardised root mean square residual (*SRMR*). Kline (2005) suggested that for *RMSEA* values below 0.08, *SRMR* equal to or less than 0.05 and *TLI* and *CFI* values greater than 0.90 are considered to be acceptable in model fitness.

**TABLE 1:** Reliability index and goodness-of-fit for the selected instruments.

		Cronbach’s alpha			Goodness-of-fit index					
Subscale	No. of items	Pretest	Posttest	Delayed posttest	χ^2^	*df*	*TLI*	*CFI*	*SRMR*	*RMSEA*
Non-verbal intelligence	60	0.82								
Metacognition test					2342.34	568	0.97	0.98	0.02	0.03
Knowledge	9	0.86	0.87	0.87						
Experience	6	0.85	0.86	0.86						
Regulation	10	0.80	0.82	0.82						
Learning motivation					732.85	297	0.90	0.92	0.05	0.08
Interest	5	0.77	0.78	0.78						
Competence	5	0.72	0.73	0.73						
Effort	5	0.65	0.69	0.69						
Evaluation	5	0.78	0.80	0.80						
Pressure	5	0.74	0.76	0.76						
Scientific ability					125.72	34	0.91	0.91	0.04	0.07
Mathematical	4	0.65	0.68	0.68						
Spatial	4	0.71	0.75	0.75						

To control the effect from the instructor (e.g., instructional style, teaching experience, and familiarity level of astronomy knowledge) on McR intervention implementation, this study randomly selected a science teacher as the instructor from the participants’ school. Furthermore, this instructor received a workshop before the intervention programme. At the workshop, one research collaborator presented a checklist to this instructor to remind what the instructor expected to do in EG, including the time arrangement for each teaching section, principles of McR word use to EG students, and the detailed information (e.g., key concept of McR) for instructional word suggestions to EG and CG students.

All participants were required to take the metacognition test, science learning motivation survey, and science thinking test, each of which with a pretest, posttest, and a 2-week delayed posttest. The metacognition test, science learning motivation survey, and science thinking test lasted for 15, 15, and 10 min, respectively. At the pretest, students took 50 and 15 min on the non-verbal intelligence test and working memory test, respectively.

#### Experimental Design

A quasi-experiment with a pretest, posttest, and a 2-week delayed treatment-control group design was applied. EG and CG received a treatment-control group design in the same digital classroom to remove the effect of available resources (e.g., astronomy model utilisation). McR intervention was inserted into the students’ astronomy learning activities for the EG group, whilst the CG students received the typical activities instruction (e.g., group discussion on what they learned) during the same time when EG students received the McR component. The difference between EG and CG is the inserted learning activity design: EG received McR practices and CG received typical group discussion practices. The learning pace, learning materials for astronomy knowledge, and the number of learning sessions were similar between EG and CG students.

#### Instructional Design

Metacognitive regulation practices for EG students included monitoring and evaluating two components. The instructor required EG students to conduct McR practices at the last 10 min of the session, whilst EG students were required to evaluate their performance of astronomy knowledge acquisition and observe whether they had already achieved the expected performance requested by the school syllabus. CG students were required to conduct group discussions on what they have learned in the current session and how to implement the astronomy knowledge into practical problem-solving. CG and EG students received no feedback on their performance at every last-minute activity. The formal intervention comprised 15 science sessions through two modules, with each module including seven to eight 45-min science lessons. The first eight contents focussed on the natural knowledge relevant to the Earth (i.e., Earth rotation), and the rest was related to astronomy knowledge teaching (i.e., the correlation amongst the Earth, Moon, and Sun). Supplementary Appendix 1 provides the list of teaching and learning content in a science subject. Supplementary Appendix 2 presents the example questions for each category of McR principles.

### Measures

#### Non-verbal Intelligence Test

A full version of Raven’s standard progressive matrices (sets A–E) was used. Each item was presented with a portion missing. Students were required to select one piece from the provided six to eight options to complete the matrix items. There were 60 items and the maximum score was 60.

#### Metacognition Ability Test

This test was modified from the math metacognition ability test in the Chinese version (Lan, 2014) to fit the context of astronomy science in this study. The test comprised three 5-Likert subscales to assess the students’ metacognition knowledge, metacognition experience, and McR. The knowledge scale had nine items, assessing the students’ awareness on the link amongst science learning outcome or production, exploration activity design, and the principles (example items: I think I can handle the science principle application on a science knowledge test and I can do well). The maximum mean score on the knowledge scale was 5. The experience scale had six items, assessing the students’ cognitive feelings and corresponding affective feeling (example item: I have a clear awareness on the difficult level evaluation of the given science subject task). The maximum mean score of the metacognition experience test was 5. The regulation score had 10 items, assessing the students’ awareness of science knowledge activity progress monitoring ability (example item: I have a clear mental guideline on how to solve the given science problems *via* separating into more specific steps). The maximum mean score of the McR test was 5.

#### Science Learning Motivation

This questionnaire was modified from the Chinese primary school students’ math learning questionnaire (Rao et al., 2000), which was validated in the previous studies in Chinese participants (Ndijuye and Rao, 2019). This questionnaire aimed to measure the students’ science learning motivation through five six-item, five-point Likert subscales: interest, competence, effort, usefulness, and pressure. The maximum mean score for each subscale was 5. Specifically, the interest scale assessed the students’ self-awareness on science subject learning interest and enjoyment (example item: I think learning science knowledge is interesting). A higher score represented a higher science learning interest. The competence scale assessed the students’ self-evaluation on science knowledge learning ability (example items: I am satisfied with my learning performance in science knowledge acquisition). A higher score represented a higher science learning competence. The effort scale assessed the students’ self-evaluation on self-intrinsic learning effort (example item: I exerted a huge amount of effort on learning science). A higher score represented a higher science learning effort. The usefulness scale assessed the students’ self-evaluation on the importance of scientific knowledge acquisition (example item: science knowledge is convenient to my lifestyle). A higher score represented the high usefulness of self-assessment on science learning. The pressure scale assessed the students’ negative emotion (anxiety and pressure) on science knowledge acquisition (example item: I have learning anxiety on the science subject). The pressure scale had an inverted score account; thus, a higher score can reflect a lower science learning pressure.

#### Scientific Ability

This test consisted of two four-item subtests: the mathematical ability test and the spatial thinking ability test. The mathematical ability test assessed the students’ habits in the mind of both algorithm principle applications on mathematical problem-solving. Any correct answer was awarded one score. The maximum score of the mathematical ability test was 4. Items in the spatial ability test can measure the students’ spatial thinking logic and abstract thinking of the object’s location. Any correct answer was awarded one score. The maximum score of the spatial ability test was 4.

## Results

To address aforementioned research questions, the section “Results” contained two components. First, it provided the descriptive analysis to provide the demographical information of participants and test the normality assumption. Second, it provided the available reason to apply repeated measures to conduct the inferential analysis.

### Descriptive Analysis

The mean score and standard deviation of non-verbal intelligence, metacognition, science learning motivation, and scientific ability across the pretest, posttest, and delayed posttest are all presented in Table 2. Moreover, the skewness and kurtosis test have shown that all indicators were within ±2, indicating that no significant outliers are included in this study (Small, 1980; Hopkins and Weeks, 1990).

**TABLE 2:** Descriptive analysis.

		Pretest		Posttest		Delayed Posttest	
Variables	Group	*Mean*	*SD*	*Mean*	*SD*	*Mean*	*SD*
Non-verbal Intelligence	EG	25.52	1.96				
	CG	25.48	1.95				
Knowledge	EG	2.21	0.09	3.82	0.19	3.84	0.19
	CG	2.21	0.09	2.24	0.17	2.27	0.22
Experience	EG	1.42	0.28	3.18	0.28	3.21	0.29
	CG	1.42	0.29	1.46	0.32	1.49	0.36
Regulation	EG	2.88	0.16	3.18	0.17	3.17	0.17
	CG	2.88	0.16	2.90	0.17	2.93	0.18
Interest	EG	2.64	0.90	3.06	0.85	3.08	0.87
	CG	2.63	0.91	2.62	0.89	2.58	0.90
Competence	EG	2.38	0.78	2.74	0.78	2.75	0.79
	CG	2.38	0.79	2.38	0.79	2.37	0.79
Effort	EG	2.57	0.84	2.98	0.84	2.98	0.84
	CG	2.55	0.85	2.55	0.85	2.55	0.85
Evaluation	EG	2.64	0.87	3.28	0.88	3.28	0.88
	CG	2.63	0.87	2.61	0.88	2.60	0.88
Pressure	EG	2.41	0.74	2.03	0.74	2.03	0.74
	CG	2.42	0.73	2.42	0.73	2.42	0.72
Mathematical	EG	1.23	1.04	3.10	0.93	3.21	0.89
	CG	1.12	0.80	1.78	0.82	1.80	0.99
Spatial	EG	1.37	1.06	3.25	0.90	3.04	1.01
	CG	1.32	1.02	1.72	1.01	1.92	1.14

*EG, experimental group; CG, control group.*

### The Effects of Metacognitive Regulation Intervention on Students’ Metacognition Ability, Science Learning Motivation, and Scientific Ability

A repeated measure analysis of variance was performed to compare the students’ metacognition ability (knowledge, experience, and regulation), science learning motivation (interest, competence, effort, evaluation, and pressure), and scientific ability (mathematical and spatial ability) across the pretest, posttest, and delayed posttest on the students’ perceived instruction across different treatment groups. The heterogeneity test shows that all selected variables had insignificant heterogeneity (*p* > 0.05). Furthermore, Mauchly’s test of sphericity showed that the Mauchly’s W were insignificant (*p* > 0.10) amongst metacognition ability, science learning motivation, and scientific ability, indicating that the heterogeneity effect was insignificant amongst metacognition ability, science learning motivation, and scientific ability (Barcikowski and Robey, 1984; Gurevitch and Chester, 1986).

Time (pretest, posttest, and delayed posttest) was set as a within-subject variable. The variable was set as a group and the dependent variables were metacognition ability, science learning motivation, and scientific ability. As shown in Table 3, the variables’ interaction effects between time and group were significant, which indicated that students had different developments on the selected variables. Therefore, a simple effect was performed.

**TABLE 3:** Results of repeated measures analysis of variance.

		Time × group		Time effect on	
		intervention effect		each group	
Variables	Group	*F*-value	Partial η^2^	*F*-value	Partial η^2^
Knowledge	EG	539.22***	0.759	2077.82***	0.96
	CG			2.15	0.02
Experience	EG	161.16***	0.708	539.36***	0.85
	CG			0.62	0.01
Regulation	EG	11.09***	0.143	51.63***	0.34
	CG			0.72	0.01
Interest	EG	5.97**	0.083	12.12***	0.11
	CG			2.87	0.03
Competence	EG	4.10**	0.058	9.59***	0.09
	CG			<0.001	<0.001
Effort	EG	3.05**	0.077	11.02***	0.10
	CG			<0.001	<0.001
Evaluation	EG	13.14***	0.168	25.89***	0.21
	CG			0.66	0.01
Pressure	EG	3.69*	0.053	7.50**	0.07
	CG			0.68	0.01
Mathematical	EG	9.22***	0.122	73.01***	0.42
	CG			8.53***	0.08
Spatial	EG	6.90***	0.094	53.27***	0.35
	CG			4.10*	0.04

*p < 0.05, **p < 0.01, ***p < 0.001; EG, experimental group; CG, control group.

Within the subject comparison, results indicated that the EG students’ metacognition ability, science learning motivation, and science ability significantly increased in the posttest and delayed posttest more than in the pretest after the intervention programme. The posttest and delayed posttest scores of metacognition ability were higher than 3 on a five-point Likert scale, which indicated that the McR intervention had a positive effect on the ADHD students’ metacognition ability development. The largest change was found in the knowledge domain, followed by experience and regulation. Whilst no significant change was found in knowledge, experience, and regulation amongst the CG students, this result indicated that the intervention design had improved the EG students’ metacognition ability across knowledge, experience, and regulation.

Significant time × group interaction effect was found in the science ability through a simple effect analysis. Both EG and CG scientific abilities had improved after receiving the intervention treatment. Specifically, the significant changes were found in EG [mathematical (*F* = 73.01, *p* < 0.001, partial η^2^ = 0.42), spatial (*F* = 53.27, *p* < 0.001, partial η^2^ = 0.35)] and CG [mathematical: (*F* = 8.53, *p* < 0.001, partialη^2^ = 0.08); spatial (*F* = 4.10, *p* < 0.05, partialη^2^ = 0.04)]. These results indicated that the astronomy knowledge curriculum had improved the students’ scientific ability.

A repeated measures analysis of variance showed that the time × group interaction effect was significant. Simple effect analysis showed that only the EG students have significantly higher scores on interest (*F* = 12.12, *p* < 0.001, partial η^2^ = 0.11), competence (*F* = 9.59, *p* < 0.001, partial η^2^ = 0.09), effort (*F* = 11.02, *p* < 0.001, partial η^2^ = 0.10), evaluation (*F* = 25.89, *p* < 0.001, partial η^2^ = 0.21), and pressure (*F* = 7.50, *p* < 0.01, partial η^2^ = 0.07) at the posttest and delayed posttest than in the pretest. These results indicated that the intervention treatment has enhanced the EG students’ science learning motivation.

## Discussion

This study has confirmed that the instructional approach of McR is effective in enhancing the ADHD students’ metacognition development, science knowledge learning motivation, and scientific ability development. This study also extends the content of DIM in astronomy knowledge education and shows that through McR interaction activities, ADHD learners can have higher metacognition abilities.

### Effectiveness of Instructional Approach of Metacognitive Regulation on Metacognition Development

Through the McR intervention design, EG students performed significantly higher metacognition scores at the posttest and delayed posttest more than in the pretest. This result was consistent with previous studies, in which the intervention design could affect the students’ metacognition development (Schunk, 2008; Hacker et al., 2019). This study was conducted under a school-based classroom teaching format. ADHD students in mainland China usually did not receive any special consideration from teachers and schools during formal school teaching and learning activities. This was a general feature for most Chinese schools. However, EG students in this study acquired great opportunities on metacognition awareness network construction. EG students have experienced initial learning process planning, evaluation, and monitoring, which satisfied the requirement of constantly monitoring the behavioural exposure and brief intervals between reinforcers (Arcia et al., 2000; DuPaul et al., 2011). This would be one key reason why the EG students performed significantly higher metacognition awareness after the intervention. The EG students’ metacognition awareness was strengthened by the application of McR principles (planning, evaluation and monitoring). As a result, the level of ADHD students’ attention problem would be reduced by the high-frequency interaction amongst ADHD students, teachers’ instructional media, and group members’ reminders.

Results have shown that metacognition has a network correlation amongst knowledge, experience, and regulation. Future studies could improve the students’ metacognition performance *via* any subcategory of metacognition. Moreover, in astronomical education, it was more difficult to improve the regulation than the other two metacognition categories (knowledge and experience). This study also indicated that the group-based interactive learning format is an appropriate format for the ADHD students’ metacognition awareness development.

### Effectiveness of Metacognitive Regulation on Science Learning Motivation

This study showed that the EG students’ science knowledge learning motivation had significantly improved after the intervention. These results were consistent with previous studies, which had shown a positive correlation between metacognition performance and learning motivation (de Boer et al., 2018; Bonfils et al., 2019). Self-determination theory (SDT, Deci and Ryan, 2008) suggested using *competence*, *autonomy*, and *social relatedness* to determine the learners’ intrinsic learning motivation. There were two potential reasons to elaborate on the positive effect of McR on *interest*, *competence*, *effort*, *usefulness*, and *evaluation*. It should be the positive effect of metacognition development. McR provided the function of self-regulation during the astronomic learning process, which had a positive effect on *evaluation* and *usefulness* (de Boer et al., 2018; Bonfils et al., 2019). During astronomy knowledge acquisition, McR provided more cues and requirements to guide ADHD students on how to handle the learning target, wherein poor learners could be led to the right track. The feedback of the learning outcome for each astronomical knowledge node would contribute to the learners’ *interest*, *effort*, and *competence* score, in which the McR had enlarged the ability of *competence* on science knowledge acquisition. Moreover, due to the three principles of McR, which were fully applied to each lesson, students became used to achieve the designed requirement automatically and the demands of *autonomy* of self-motivation improvement would be satisfied.

An alternative reason was the limitation of China’s learning condition on the ADHD students’ learning performance, which could be solved by the McR design. Due to the astronomical knowledge which usually contributed a small percentage in overall academic performance evaluation, students spent less time on astronomical subject exploration. The unique learning interest of activities might not be found in traditional learning curricula. In this study, through three principle applications of McR, the students paid more time on learning under a group-based learning mode. More immersion time in science knowledge would increase the opportunity to find science learning interests (de Boer et al., 2018). Moreover, the McR design not only encouraged ADHD students to experience the discovery learning process and cultivate their learning interest, but also reduced the restriction of tangible resource requirements (e.g., computers, appropriate learning materials) and intangible resources (e.g., high professionalism of teaching science). Students could perform at a higher proficiency in science, which might result in higher learning interest (de Boer et al., 2018). Results indicated that the key to improve the ADHD students’ learning motivation was to satisfy the students’ personal intrinsic requirement in learning.

### Effectiveness of Metacognitive Regulation Design on Scientific Abilities Development

This study had shown that all students performed significantly higher scientific abilities at the posttest, echoing the suggestion that learning science could enhance the learners’ scientific abilities (Lau and Roeser, 2002; Lin and Schunn, 2016). Previous studies had shown that science literacy exposure and relevant awareness activities (e.g., cooperate thinking activities) could enhance the learners’ scientific abilities (Cherroret et al., 1992; Green et al., 2017). During astronomical knowledge learning, the curriculum not only provided science knowledge exposure to students, but also required students to think on the reason behind phenomena through various brainstorming activities. Moreover, the astronomical knowledge curriculum required learners to apply mathematical knowledge to solve problems and think about the relative locations amongst the celestial bodies, which provided a possibility for students to enhance their mathematical and spatial thinking abilities (De Backer et al., 2016; Hadwin et al., 2017). Results indicated that the astronomy learning context was an appropriate content to implement McR intervention to improve the students’ scientific abilities, whilst McR intervention enhanced the students’ awareness of monitor and control on scientific academic performances.

### Limitations and Future Directions

This study did not distinguish the effect of each McR principle on metacognition awareness, science learning motivation, and scientific abilities. For future studies, it could further specify the McR principle into more detailed categories and test every detailed McR principle factors on students’ academic achievement. Moreover, this study focussed on the effect of McR intervention on the ADHD students’ astronomy knowledge acquisition and learning motivation. Whether the key concepts in astronomy or measures of the students’ astronomy knowledge had an improvement remains unclear. For future studies, it should include more students’ achievement variables as parameter to examine the intervention effect on these learning factors. Next, this study only tested the effectiveness of McR intervention on ADHD students. For other special education needs categories (e.g., ASD), the effect of McR on these students’ academic performance remained unclear. For future studies, it should test the possibility of McR intervention on students with different special education needs. Finally, although the same instructor was trained separately regarding the two conditions, the instructor still may potentially carry over some intervention elements to the control condition, and the possible risk would affect the results of CG students’ learning abilities. For future studies, it could take online experimental design to treat both EG and CG students at the same time, and the instruction to students should be fully controlled and presented online. The effect of online intervention format should be further explored.

## Conclusion

This study provides evidence that the McR intervention design can improve the ADHD learners’ scientific ability development, science knowledge learning, and overall metacognition development. Moreover, the dynamic interaction model is appropriate in providing sufficient guidelines for ADHD students to learn astronomical knowledge. The interaction activities amongst peers, teacher, and students are shown to benefit students’ knowledge acquisition in astronomy.

## Data Availability Statement

The raw data supporting the conclusions of this article will be made available by the authors, without undue reservation.

## Ethics Statement

The studies involving human participants were reviewed and approved by the Guangzhou University. Written informed consent to participate in this study was provided by the participants’ legal guardian/next of kin.

## Author Contributions

HYZ contributed data collection and dataset construction. YD contributed data analysis and draft writing. YKS contributed idea in research design and draft writing. JY contributed the revision on research design. CBY contributed the revision of data analysis and draft writing. JDW and WYD contributed draft writing. All authors contributed to the article and approved the submitted version.

## Conflict of Interest

The authors declare that the research was conducted in the absence of any commercial or financial relationships that could be construed as a potential conflict of interest.

## Publisher’s Note

All claims expressed in this article are solely those of the authors and do not necessarily represent those of their affiliated organizations, or those of the publisher, the editors and the reviewers. Any product that may be evaluated in this article, or claim that may be made by its manufacturer, is not guaranteed or endorsed by the publisher.
