# Supplementary material for: Effectiveness of Metacognitive Regulation Intervention on Attention-Deficit–Hyperactivity Disorder Students’ Scientific Ability and Motivation
Source: Front Psychol. 2021 Dec 23;12:747961. doi: 10.3389/fpsyg.2021.747961 (PMC8732764; doi:10.3389/fpsyg.2021.747961)
Supplement: Supplementary file 1 [file Data_Sheet_1.pdf]

**Appendix I**

Learning Content for 16 Science Lessons

| Module I Earth Knowledge | Content |
|---|---|
| 1 | 昼夜交替现象( Alternating day and night phenomenon) |
| 2 | 人类认识地球及其运动的历史(Human understanding of the history of the earth and its movement) |
| 3 | 证明地球在自转(Prove the Earth's rotation) |
| 4 | 谁先迎来黎明(Who will usher in the dawn first) |
| 5 | 北极星“不动”的秘密(The secret of Polaris "not moving" ) |
| 6 | 地球在公转吗(Is the earth in orbit) |
| 7 | 为什么一年有四季(Why there are four seasons a year) |
| 8 | 极昼和极夜的解释(Explanation of polar day and night) |
| Module 2 Astronomy Knowledge | |
| 1 | 地球的卫星-月球(Earth's satellite-Lunar) |
| 2 | 月相变化(Lunar phase changes) |

| | |
|---|---|
| 3 | 我们来造环形山(Let's build a crater) |
| 4 | 日食和月食(Solar and lunar eclipses) |
| 5 | 太阳系(Solar system) |
| 6 | 在星空中(In the stars) |
| 7 | 探索宇宙(Explore the universe) |

**Appendix II**

*Example Items for each Category of Metacognition Reflection Principles*

| Principles | Category | Example Guideline on Worksheet |
| --- | --- | --- |
| | Monitoring | 1. The track assessment of the learning goals<br>2. The situation of your learning progress in the overall learning procedure<br>3. Adjustment method to adjust the learning activity |
| | Evaluating | 1. Performance evaluation in the learning activity<br>2. The assessment on study product<br>3. The study design is acceptable for the following reasons |
